# Between the hype and harm: does artificial intelligence in health offer solace or further exclusion for marginalised populations in Sub-Saharan Africa? A scoping review

**DOI:** 10.1080/16549716.2026.2710547

**Published:** 2026-08-03

**Authors:** Talent Tapera, Tennyson Mgutshini

**Affiliations:** College of Graduate Studies, University of South Africa, Pretoria, South Africa

**Keywords:** Artificial intelligence, health equity, digital health, healthcare disparities, marginalised populations

## Abstract

Artificial intelligence (AI) is increasingly being integrated into healthcare systems and has the potential to improve health outcomes. In Sub-Saharan Africa (SSA), however, concerns remain that AI may either reduce or exacerbate existing health inequities depending on how it is developed, governed, and implemented. This scoping review aimed to map and synthesise the existing evidence on the implications of AI for health equity among marginalised populations in Sub-Saharan Africa. PubMed, Web of Science, Scopus, and selected grey literature sources were searched between February and March 2026. Peer-reviewed and grey literature examining AI applications, governance, or implementation in healthcare involving marginalised populations or health systems within SSA were eligible for inclusion. The review followed the Arksey and O’Malley methodological framework and the PRISMA-ScR reporting guideline. Two reviewers independently screened sources of evidence and extracted data using a standardised charting form, and findings were synthesised thematically. Twenty-three sources of evidence met the inclusion criteria. Two dominant narratives emerged. AI may reinforce existing inequities through digital infrastructure gaps, algorithmic bias, under-representation of African datasets, weak governance, and data colonialism. Conversely, AI has the potential to improve health equity by expanding healthcare access, strengthening disease surveillance, supporting health system planning, and improving access to specialised services. Across the literature, AI’s impact consistently depended on equitable infrastructure, inclusive governance, and context-specific implementation. AI has considerable potential to advance health equity in SSA. However, achieving equitable benefits requires investment in digital infrastructure, representative data systems, ethical governance, and inclusive policies.

## Paper Context

This paper situates AI within the context of health equity in Sub-Saharan Africa.

**Main findings:** Artificial intelligence in African health systems presents both opportunities to expand healthcare access and risks of reinforcing existing inequalities driven by infrastructure gaps, data bias, and governance challenges.

**Added knowledge:** This review provides a focused synthesis of how artificial intelligence specifically affects marginalised populations in Sub-Saharan Africa, highlighting the structural conditions that shape equitable or inequitable outcomes.

**Global health impact for policy and action:** The findings emphasise the need for deliberate policy action on inclusive data systems, digital infrastructure investment, and contextually grounded governance to ensure artificial intelligence advances health equity rather than deepening disparities.

## Background

Over recent years Artificial intelligence (AI) has emerged as a highly promising innovation in various sectors including global health. It has been well documented that it has potential to enhance diagnostics, streamline clinical decision-making [1,2]. However, the extent to which AI meaningfully expands constrained health resources in low- and middle-income settings remains insufficiently understood. AI systems depend on large, high-quality datasets for training and validation. Yet most health datasets used to train algorithms are sourced from high-income countries, leading to limited representation of African populations and health conditions [3]. Moreso, the ethical and normative foundations of AI governance are predominantly shaped by frameworks developed in Western contexts [4]. As such, this mismatch can lead to governance mechanisms that fail to protect marginalised populations or to confront historical and structural forms of exclusion embedded in health systems in Sub-Saharan Africa.

Across Africa, emerging empirical research highlights both optimism about AI’s capacity to improve health service delivery and deep concerns about fairness, bias, and implementation barriers. Studies find that while general populations may express trust in AI, experts raise concerns about ethical implications, fairness indicators, and systemic obstacles to equitable integration and governance [5]. Policy analyses and stakeholder reports further emphasise that without proactive, equity-focused policy measures AI risks reinforcing ‘digital colonialism’ where technologies developed outside local contexts are imposed in ways that undermine local priorities and marginalised groups’ access to care [3].

Artificial intelligence (AI) is increasingly presented as a transformative technology capable of improving healthcare delivery and strengthening health systems. However, the implications of these technologies for marginalised populations within Sub-Saharan Africa remain insufficiently understood. Artificial intelligence has the potential to increase access to healthcare and improve disease surveillance, but there are concerns that the use of technology may reproduce or exacerbate existing social and structural inequalities if data representation, infrastructure, and governance are not addressed. This scoping review, therefore, aimed to synthesise existing evidence on the role of artificial intelligence in shaping health equity in African health systems. To guide this review, the following research questions were developed:

- How do artificial intelligence applications influence health equity for marginalised populations in Sub-Saharan Africa?
- What systemic, structural, and governance challenges affect the equitable deployment of AI technologies in health systems in SSA?
- How are issues related to algorithmic bias, data representation, and digital infrastructure addressed within the existing literature on AI and health in SSA?

## Methods

Our scoping review design was guided by the methodological frameworks proposed by Arksey and O’Malley [6]. The objectives of conducting a scoping review, as outlined by Arksey and O’Malley, were achieved by providing a comprehensive summary of existing research on AI in health as it related marginalisation in SSA also by identifying any gaps present in the current literature. This protocol follows the reporting guidelines of the Preferred Reporting Items for Systematic Reviews and Meta-Analysis for scoping review (PRISMA-ScR) [7]. This scoping review was registered in Open Science Framework prospectively with Project DOI: 10.17605/OSF.IO/3P46T (accessible via: https://osf.io/3p46t/overview).

The eligibility criteria for this scoping review were developed to identify sources of evidence examining the role of artificial intelligence (AI) in relation to marginalised populations within Sub-Saharan African health systems. Using the Population, Concept, Context framework commonly applied in scoping reviews, sources of evidence were included if they focused on marginalised populations within Sub-Saharan Africa (Population) and examined AI-enabled health interventions (Concept), including diagnostic tools, clinical decision-support systems, patient risk stratification, drug discovery, disease surveillance systems, and health service management applications such as scheduling optimisation. Sources of evidence conducted in any healthcare setting within Sub-Saharan Africa were eligible for inclusion, including public health systems, private healthcare facilities, community-based services, and tertiary healthcare institutions (Context).

The review included empirical studies employing qualitative, quantitative, or mixed methods designs, as well as conceptual analyses, policy reports, technical papers, and documented case studies that examined the application or governance of AI technologies in African health systems. Given the rapidly evolving nature of artificial intelligence in global health, grey literature sources were also included to capture emerging evidence, policy developments, and programmatic experiences that may not yet be represented in peer-reviewed publications. Grey literature included reports from international organisations, policy institutions, and recognised research initiatives focusing on AI in health across Africa.

## Exclusion criteria

**Figure 1.**
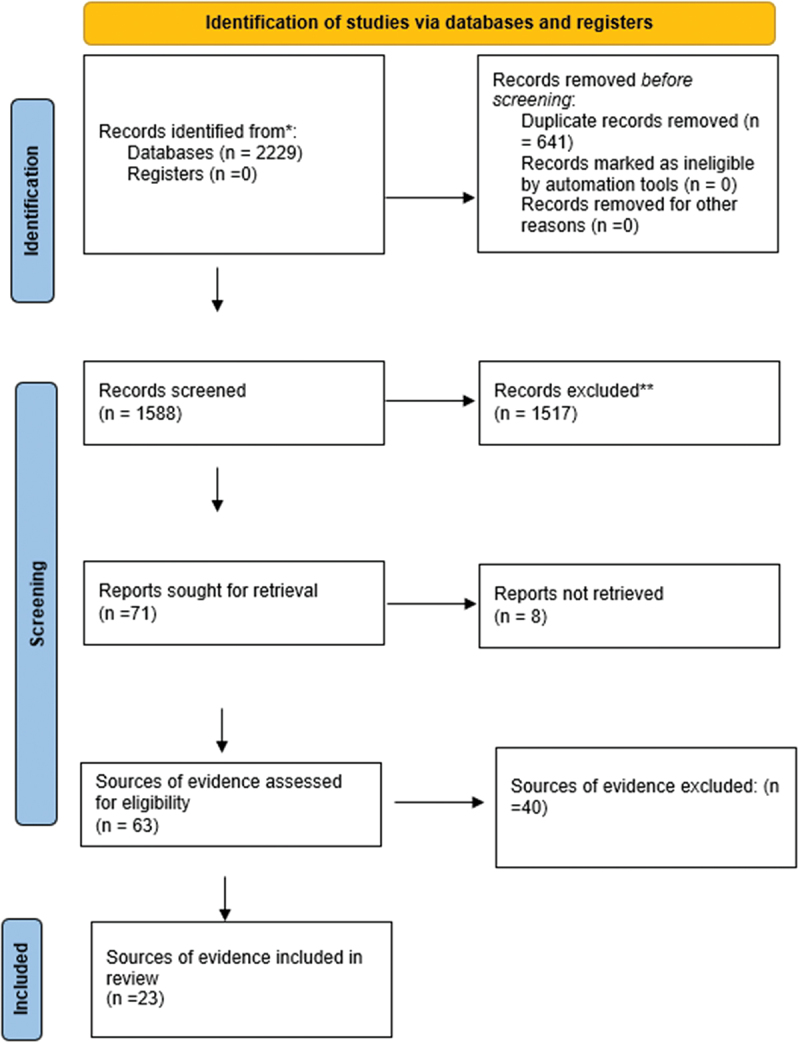
PRISMA 2020 flow diagram illustrating the identification, screening, eligibility assessment, and inclusion of sources of evidence in this scoping review.

Sources of evidence were excluded if they: (i) focused solely on technical algorithm development without application to healthcare or health systems; (ii) examined artificial intelligence outside the health sector; (iii) were conducted outside Sub-Saharan Africa; (iv) did not address health equity, marginalised populations, artificial intelligence governance, implementation, or health system applications; or (v) were not available in English. These eligibility criteria were applied during title screening, abstract screening, and full-text review, as illustrated in the PRISMA 2020 flow diagram (Figure 1).

## Data synthesis

Data from the included sources were synthesised using a thematic synthesis approach to integrate findings from both qualitative and quantitative evidence. The analytical process followed three stages commonly used in qualitative synthesis. First, the findings, discussion, and conclusions sections of each included study were systematically reviewed, and initial codes were generated inductively, allowing themes to emerge directly from the data rather than being predetermined. Second, common codes were combined into descriptive themes that represented recurrent themes in the literature on the role of artificial intelligence in health equity in African contexts. These descriptive themes captured issues related to digital infrastructure, algorithmic bias, data governance, access to healthcare, and health system strengthening. Third, these descriptive themes were further interpreted and synthesised into higher-order analytical themes that addressed the core research questions guiding the review. The final themes, therefore, reflected both the risks and opportunities associated with the deployment of AI technologies in African health systems, particularly in relation to marginalised populations.

## Search strategy

### Electronic databases

Electronic database searches were conducted in PubMed, Web of Science, and Scopus. In addition, grey literature was identified through targeted searches of organisational websites, policy reports, and recognised research initiatives relevant to AI and health in Africa. Language or geography restrictions were applied to the search to be limited to English and Sub-Saharan Africa, respectively. Database searches were conducted between February and March 2026

### Study selection

Firstly, we performed extensive title screening by searching and uploading all literature search results on a reference management software library, Mendeley. Secondly, we deduplicated sources from the library, leaving only unique sources of evidence. Thirdly, we conducted an extensive abstract screening to determine inclusion in a full-text review. At this stage, records that did not meet the predefined eligibility criteria described in the ‘Eligibility Criteria’ section were excluded from further consideration.

### Search strategy

Search terms were organised into four concept blocks representing (i) artificial intelligence, (ii) health, (iii) health equity and marginalisation, and (iv) Sub-Saharan Africa. Controlled vocabulary (e.g. MeSH terms) and free-text terms were combined using Boolean operators (AND/OR). The complete PubMed search strategy, including field tags, Boolean operators and search limits, is provided in Supplementary Appendix 2. Equivalent search strategies were adapted for Web of Science, Scopus, and grey literature sources using database-specific syntax while retaining the same conceptual structure.

Search terms used combined four concept block as below;

1. Artificial Intelligence
  (‘Artificial Intelligence’ OR ‘Machine Learning’ OR ‘Deep Learning’ OR ‘Algorithm*’ OR ‘Predictive model*’ OR ‘Neural network*’)
2. Health
  (‘Health’ OR ‘Healthcare’ OR ‘Digital health’ OR ‘mHealth’ OR ‘eHealth’ OR ‘Clinical decision support’)
3. Equity and Marginalisation
  (‘Equity’ OR ‘Inequality’ OR ‘Inequity’ OR ‘Marginali*’ OR ‘Vulnerable population*’ OR ‘Access’ OR ‘Exclusion’ OR ‘Bias’ OR ‘Justice’ OR ‘Discrimination’ OR ‘Stigma’ OR ‘PROGRESS-Plus’)
4. Africa
  (‘Sub-Saharan Africa’ OR ‘SSA’ OR ‘Africa South of the Sahara’)

The full search strategy was adapted for each database.

### Data charting

A standardised data-charting form was developed by the review team and pilot-tested on a sample of eligible sources of evidence to ensure consistency and completeness before full data extraction. The form was refined iteratively following the pilot exercise.

Two reviewers independently charted data from all included sources of evidence using the standardised form. Any discrepancies identified during the charting process were resolved through discussion and consensus between the reviewers. Where necessary, the review team revisited the original source to verify extracted information and ensure accuracy.

The following data items were extracted from each included source: (i) bibliographic information (author(s), year of publication, and publication type); (ii) country or geographical setting; (iii) study aim or objective; (iv) study design or source type; (v) healthcare setting; (vi) artificial intelligence application or technology described; (vii) target population; (viii) health condition or health system focus; (ix) equity or marginalisation issues addressed; (x) governance and ethical considerations; (xi) key findings; and (xii) implications for health equity in Sub-Saharan Africa.

Consistent with the methodological guidance of Arksey and O’Malley, and the PRISMA-ScR reporting guideline, the purpose of data charting was to systematically map the breadth and characteristics of the available evidence rather than to evaluate intervention effects or measure reviewer performance. Thus, formal inter-rater reliability statistics, such as Cohen’s kappa, were not calculated. Instead, discrepancies were resolved through discussion and consensus, which is an accepted approach for scoping reviews.

### Critical appraisal of sources of evidence

Consistent with the objectives of this scoping review and established methodological guidance for scoping reviews, a formal critical appraisal of the included sources was not undertaken. The purpose of this review was to map the breadth, characteristics, and nature of the available evidence on artificial intelligence and health equity in Sub-Saharan Africa rather than to evaluate the methodological quality of individual sources of evidence. Nevertheless, the findings were interpreted with consideration of the diversity of evidence sources, including peer-reviewed publications and grey literature, and this is acknowledged as a limitation of the review.

## Results

### Characteristics of included sources

A total of 23 sources of evidence met the eligibility criteria and were included in this scoping review. The included evidence comprised four empirical studies (17.4%), nine review, commentary, and perspective papers (39.1%), six conceptual and ethics-focused papers (26.1%), and four policy reports or grey literature sources (17.4%). The diversity of evidence reflects the emerging nature of research on artificial intelligence and health equity in Sub-Saharan Africa, where scholarly output remains dominated by conceptual discussions, policy analyses, and expert perspectives, with comparatively fewer empirical studies evaluating AI implementation in real-world health settings.

### Geographical distribution of included sources

The included sources represented a range of geographical contexts across Sub-Saharan Africa and the broader African continent. Country-specific evidence was identified from South Africa, Nigeria, Uganda, Kenya, Zimbabwe, Lesotho, Ghana, and Rwanda, while several sources adopted a broader Sub-Saharan African, Africa-wide, or multi-country African perspective. Despite this geographical diversity, the evidence was concentrated in a relatively small number of countries, particularly South Africa and Nigeria, with limited published evidence from Central Africa and several low-income countries within Sub-Saharan Africa. This uneven geographical distribution highlights important knowledge gaps and suggests that the current evidence base may not fully reflect the diversity of health systems and implementation contexts across the region.

The thematic synthesis of the included sources generated several analytical themes describing both the opportunities and risks associated with the deployment of artificial intelligence in African health systems. These themes capture key issues related to infrastructure, governance, algorithmic bias, healthcare access, and health system strengthening. Table 1 presents the identified themes from the review and the sources that contributed to each theme.

**Table 1.** Thematic mapping.

Theme	Description	Sources Contributing
Digital Infrastructure Gaps and Risk of Exclusion	Sources highlighting how inadequate digital infrastructure, unreliable internet connectivity, electricity shortages, workforce capacity constraints, and technological readiness influence the adoption and equitable implementation of AI-enabled health technologies in Sub-Saharan Africa.	Janneker (2025); Asiedu et al. (2025); Alaran et al. (2025); Owoyemi et al. (2020); Monethi (2024); Kyambade & Namatovu (2025); Malope (2025); Umah et al. (2025); AI in Zimbabwean Healthcare (2025)
Algorithmic Bias, Data Inequality and Representation	Sources describing how limited African datasets, under-representation of African populations, data quality issues, and algorithmic design contribute to biased AI models and inequitable healthcare outcomes.	Asiedu et al. (2024); Asiedu et al. (2025); Ugar & Malele (2024); Owoyemi et al. (2020); Responsible AI in Africa (2023); Ochasi et al. (2025); Grancia (2025)
Governance, Ethics and Data Colonialism	Sources examining ethical concerns, governance challenges, regulatory preparedness, data sovereignty, data extraction, colonial power imbalances, accountability, and culturally responsive AI governance frameworks.	Science for Africa Foundation (2025); Science for Africa Foundation (Nigeria Policy Brief, 2025); De Matas (2025); Grancia (2025); Ochasi et al. (2025); Responsible AI in Africa (2023); Monethi (2024); Adunimay (2025)
Expanding Access to Healthcare for Marginalised Populations	Sources demonstrating how AI-enabled digital health technologies, telemedicine, decision-support systems, virtual assistants, and mobile health platforms can improve access to healthcare among underserved populations.	Qoseem et al. (2024); Malope (2025); Adunimay (2025); Olatokun & Rampanyane (2025); AI in Zimbabwean Healthcare (2025); Janneker (2025); Umah et al. (2025)
Strengthening Disease Surveillance, Diagnosis and Health System Performance	Sources describing the application of AI for disease surveillance, clinical diagnosis, predictive analytics, cancer care, health system planning, and improved healthcare decision-making.	Akingbola et al. (2024); Owoyemi et al. (2020); Asiedu et al. (2025); Olatokun & Rampanyane (2025); Science for Africa Foundation (Nigeria Policy Brief, 2025); Umah et al. (2025)
Mental Health, Trust and Community-Centred AI	Sources discussing AI applications for mental health, culturally appropriate AI systems, trust, public perceptions, participatory AI development, and African-centred approaches such as Ubuntu.	Ugar & Malele (2024); Kyambade & Namatovu (2025); Asiedu et al. (2025); Ochasi et al. (2025); De Matas (2025)

## Digital infrastructure gaps and the risk of exclusion

While AI-enabled healthcare systems offer considerable potential, the reviewed sources consistently identified digital infrastructure as one of the major barriers to equitable AI implementation in Sub-Saharan Africa. Unequal access to reliable internet connectivity, stable electricity supply, digital technologies, and technical capacity was frequently reported as limiting the adoption of AI-enabled healthcare services, particularly in rural and underserved communities [5,8]. Without targeted investments in digital infrastructure, AI innovations may disproportionately benefit populations that already have better access to technology. Several sources further demonstrated how these infrastructural barriers affect AI implementation in practice. For example, healthcare leaders in Uganda identified inadequate digital infrastructure, workforce capacity constraints, and weak institutional readiness as major obstacles to integrating AI into routine healthcare delivery, while evidence from Nigeria similarly highlighted inadequate infrastructure, limited technical expertise, and insufficient funding as key barriers to AI adoption [8–10]. Collectively, the reviewed sources indicate that these structural challenges continue to constrain the equitable implementation of AI technologies across African health systems [11].

## Algorithmic bias and data inequality

Another important issue identified across the reviewed sources relates to the representativeness of the datasets used to train AI algorithms. Many AI systems continue to be developed using datasets derived predominantly from high-income countries [9,12]. When applied in low- and middle-income settings without adequate contextual adaptation, these models may produce inaccurate predictions or inequitable outcomes. Several sources highlighted that the limited inclusion of African populations in AI training datasets increases the risk of reduced diagnostic accuracy and inappropriate clinical decision-making for local populations.

The reviewed sources further demonstrated that algorithmic bias may arise at multiple stages of the AI development pipeline, including data collection, model training, and validation. For example, studies emphasised that the under-representation of African demographic, genetic, and epidemiological data can compromise the performance of AI-enabled clinical decision-support systems, while limited involvement of African researchers in AI development may perpetuate biases embedded within existing datasets [5,13]. Collectively, the evidence highlights the importance of developing locally representative datasets and promoting greater African participation in AI research and development to improve fairness, transparency, and equitable health outcomes.

## Governance challenges and data colonialism

The governance of AI technologies in global health emerged as another important theme across the reviewed sources. Several sources highlighted that many AI-enabled health technologies deployed in African countries are developed by institutions based in high-income countries, creating concerns about power asymmetries in global health innovation and digital governance. The reviewed evidence described how health data generated in low- and middle-income countries may be used to develop AI technologies without equitable sharing of benefits, a phenomenon referred to as data colonialism [4]. Such practices have the potential to reinforce existing global inequalities in knowledge production, technology development, and decision-making [13,14].

The reviewed sources also identified the need for governance frameworks that are responsive to African contexts. For example, Ubuntu-inspired ethical frameworks were proposed to promote fairness, accountability, collective participation, and culturally grounded AI governance [15]. Similarly, critical reflections on AI ethics argued that governance systems should strengthen data sovereignty, encourage meaningful participation of African stakeholders, and ensure that AI technologies are developed and implemented in ways that reflect local priorities and values [13–15].

## Expanding access to healthcare for marginalised populations

The reviewed sources consistently highlighted the potential of AI to improve access to healthcare services for populations that have historically experienced barriers to care. AI-enabled technologies, including clinical decision-support systems, telemedicine platforms, virtual health assistants, and predictive analytics, were identified as promising approaches for extending healthcare services to underserved and remote communities where shortages of healthcare professionals and limited access to specialised services remain common [16–18].

Several sources provided practical examples of these opportunities. In South Africa, AI-supported e-health technologies were reported to enhance remote consultations and facilitate more timely healthcare delivery, while evidence from Lesotho highlighted the potential of AI-driven diagnostic systems to improve disease detection and clinical decision-making in resource-constrained settings [17,18]. More broadly, the reviewed sources suggested that when implemented within appropriate governance and infrastructure frameworks, AI technologies have the potential to improve healthcare accessibility, efficiency, and continuity of care for marginalised populations across Sub-Saharan Africa [16–19].

## Strengthening disease surveillance, diagnosis, and health system performance

The reviewed sources identified AI as having considerable potential to strengthen disease surveillance, clinical diagnosis, and overall health system performance across Sub-Saharan Africa. AI applications were reported to support early disease detection, clinical decision-making, predictive analytics, and more efficient allocation of healthcare resources, thereby enhancing the responsiveness of health systems [20–26].

Several sources provided practical examples of these applications. AI-enabled diagnostic tools were highlighted as improving the early detection and management of diseases, including cancer, while predictive analytics and machine learning algorithms were recognised for their potential to support disease surveillance and health system planning [20,21,27]. Policy reports further emphasised that integrating AI into routine health information systems could strengthen evidence-based decision-making and improve service delivery, provided that appropriate governance, infrastructure, and workforce capacity are in place [22,23]. Collectively, the reviewed evidence suggests that AI can contribute to more efficient and responsive health systems when implemented within supportive policy and institutional environments.

## Improving access to mental health and specialised services

The reviewed sources highlighted the growing potential of AI to improve access to specialised healthcare services, particularly in mental health, where shortages of trained professionals remain a significant challenge across Sub-Saharan Africa. AI-enabled technologies, including chatbots, automated counselling systems, and machine learning models, were identified as promising approaches for expanding access to psychological support and specialist services in resource-constrained settings [28,29].

Practical examples from the reviewed evidence demonstrated how AI applications could complement existing health services. In Uganda, AI models developed using mental health helpline conversations were reported to identify individuals at risk of psychological distress and facilitate the provision of automated support in local languages, thereby extending access to mental health services among underserved populations [28]. Other sources similarly emphasised that culturally appropriate AI systems, developed using locally relevant data and ethical frameworks, could improve the accessibility and acceptability of specialised healthcare while helping to address persistent shortages of mental health professionals across the region [29].

## Discussion

To contextualise our findings within the broader global literature on AI and health equity, we also draw on evidence from studies conducted outside Sub-Saharan Africa.

### Artificial intelligence as a transformative opportunity for health equity

The findings of this review indicate that artificial intelligence has considerable potential to strengthen health systems and advance health equity across Sub-Saharan Africa when implemented within supportive policy, governance, and infrastructure environments. Across the reviewed sources, AI was consistently associated with opportunities to improve disease surveillance, diagnostic accuracy, health system planning, and access to healthcare through digital health technologies. Collectively, these findings suggest that AI should be viewed not merely as a technological innovation but as a potential enabler of more equitable healthcare delivery, particularly in settings where shortages of healthcare workers, geographical barriers, and limited specialist services continue to constrain access to care.

These findings are consistent with the broader global literature, which increasingly recognises AI as a tool for strengthening health systems through enhanced clinical decision support, predictive analytics, and digital service delivery [30–36]. The African evidence reviewed in this study similarly demonstrates that AI-enabled telemedicine platforms, clinical decision-support systems, mobile health applications, and predictive models have the potential to improve healthcare accessibility and efficiency. Importantly, however, the review also demonstrates that these benefits are unlikely to be realised automatically. Rather, their successful implementation depends on parallel investments in digital infrastructure, workforce capacity, governance, and locally relevant AI development.

### Risks of reinforcing existing inequalities

While artificial intelligence offers considerable opportunities to strengthen health systems, the findings of this review also demonstrate that its benefits are unlikely to be equitably realised without deliberate attention to existing structural inequalities. Across the included sources, inadequate digital infrastructure, limited digital literacy, under-representation of African populations in training datasets, and weak governance frameworks emerged as interconnected challenges that may constrain equitable AI implementation. Rather than creating new forms of inequity, AI may amplify pre-existing disparities if technologies are developed and deployed within already unequal health systems.

These findings align with growing international evidence demonstrating that AI systems are not inherently neutral but instead reflect the characteristics and assumptions embedded within the data, algorithms, and governance structures from which they are developed [37,38]. For African health systems, this raises concerns because many AI technologies continue to be developed using datasets and design approaches that inadequately represent African populations. In turn, algorithmic bias may reduce diagnostic accuracy, limit the relevance of AI-supported clinical decision-making, and inadvertently exclude already marginalised communities. These findings reinforce the importance of promoting locally representative datasets, strengthening data governance, and ensuring meaningful participation of African researchers, policymakers, healthcare professionals, and communities throughout the AI development lifecycle.

Collectively, these findings suggest that the equitable implementation of artificial intelligence requires addressing the structural barriers identified across the included sources. Without adequate safeguards, artificial intelligence may reinforce rather than reduce existing health inequities in Sub-Saharan Africa.

#### Conditions for equitable AI implementation

The evidence reviewed in this study suggests that the impact of AI on health equity is largely contingent on the broader social, institutional, and governance contexts in which these technologies operate. AI technologies can provide powerful tools to improve healthcare delivery, but benefits are not automatically distributed across populations. Equitable outcomes require intentional efforts to overcome structural barriers to AI adoption. Investments in digital infrastructure, workforce training, and data governance are key to ensure that AI technologies can be effectively integrated into healthcare systems. Without such investments, AI technologies risk being out of reach for populations that stand to benefit the most from them. Participatory, community-based approaches to AI development are equally important. Involving healthcare workers, policymakers, and local communities in the development and deployment of AI technologies can help to make sure that these systems are responsive to local needs and contexts. Participatory approaches can also help build trust in AI technologies and support their adoption in healthcare systems. Finally, we need to establish stronger regulatory frameworks for the use of AI in healthcare. Clear policies on data protection, transparency of algorithms and accountability will be key to the responsible and ethical deployment of AI technologies.

#### Implications for global health

This review adds to the ongoing global discussion around the role of AI in advancing health equity. AI technologies hold great promise to enhance the delivery of healthcare and to strengthen health systems, but their impact on health equity will be highly dependent on how such technologies are designed, governed, and implemented. These findings underscore for policymakers the importance of embedding AI technologies into broader health system strengthening strategies. AI should not be viewed as a silver bullet but rather as part of a holistic approach to improving access to healthcare and reducing health disparities. Future studies need to examine the impact of AI technologies on health outcomes and equity in real-world settings. Although many studies have proposed the potential benefits of AI, there is little empirical evidence on how these technologies impact access and outcomes in healthcare for different population groups.

## Critical reflections on the evidence base

This review demonstrates that the evidence base on AI and health equity in Sub-Saharan Africa remains at an early stage of development. Although the included sources consistently recognised the potential of AI to strengthen health systems and improve healthcare access, much of the current literature is conceptual, comprising reviews, perspectives, ethics papers, and policy analyses, with comparatively few empirical studies evaluating the implementation and impact of AI within routine healthcare settings. As a result, much of the available evidence reflects anticipated opportunities and challenges rather than measured health outcomes.

This finding highlights an important research gap. Future studies should move beyond conceptual discussions to generate robust empirical evidence on the effectiveness, safety, cost-effectiveness, acceptability, and equity impacts of AI-enabled health interventions in diverse African settings. Such evidence will be essential for informing policy, guiding responsible implementation, and ensuring that AI technologies contribute meaningfully to strengthening health systems and reducing health inequities. Greater investment in implementation research, locally led innovation, and interdisciplinary collaboration will also be critical for developing AI solutions that are responsive to African health priorities and contexts [39,40].

## Strengths and limitations

This scoping review has several strengths. To our knowledge, it is among the first reviews to comprehensively synthesise evidence on the implications of artificial intelligence for health equity in Sub-Saharan Africa. By incorporating both peer-reviewed and grey literature, the review provides a broad overview of the emerging evidence, capturing not only empirical research but also policy documents, conceptual papers, and expert perspectives that are particularly important within this rapidly evolving field. The use of a transparent and systematic scoping review methodology further enhances the credibility and reproducibility of the review process.

Nevertheless, several limitations should be acknowledged. First, the available evidence remains limited and is dominated by conceptual, commentary, and policy-oriented publications, with relatively few empirical studies evaluating AI implementation within African health systems. Second, only English-language sources published between 2020 and 2025 were included, which may have resulted in the exclusion of relevant studies published in other languages or outside the specified period. Finally, as a scoping review, this study aimed to map and synthesise the available evidence rather than formally assess methodological quality or estimate the effectiveness of AI interventions. Therefore, the findings should be interpreted as an overview of the current evidence landscape rather than definitive evidence of effectiveness.

## Implications for policy and research

The findings of this review have several implications for policy, practice, and future research. First, governments and health policymakers should ensure that AI is integrated into broader digital health strategies through governance frameworks that prioritise health equity, transparency, accountability, and data sovereignty. Investments in digital infrastructure, workforce development, and digital literacy will be essential to support the equitable implementation of AI across diverse healthcare settings. Policies should also encourage the development and validation of AI technologies using locally representative datasets to minimise algorithmic bias and improve their relevance for African populations.

Second, stronger collaboration between governments, researchers, healthcare providers, and technology developers will be necessary to promote responsible AI innovation that reflects local health priorities and community needs. Meaningful engagement of African researchers and institutions throughout the AI development lifecycle may contribute to more contextually appropriate, culturally responsive, and sustainable AI solutions.

Finally, future research should prioritise implementation and evaluation studies that assess the effectiveness, acceptability, cost-effectiveness, and equity impacts of AI-enabled health interventions in real-world African settings. Expanding the empirical evidence base will be critical for informing policy decisions and ensuring that AI contributes to reducing, rather than reinforcing, existing health inequities.

## Conclusion

Artificial intelligence has the potential to transform healthcare delivery and contribute to improved health equity across Sub-Saharan Africa by strengthening disease surveillance, supporting clinical decision-making, expanding access to healthcare, and improving health system planning. However, the findings of this review demonstrate that these potential benefits are unlikely to be realised equitably without deliberate efforts to address persistent structural challenges, including inadequate digital infrastructure, limited digital literacy, algorithmic bias, weak governance frameworks, and the under-representation of African populations in AI development.

The review further highlights that the current evidence base remains largely conceptual, with comparatively few empirical studies evaluating the implementation and equity impacts of AI in African health systems. Addressing this evidence gap will require greater investment in implementation research, locally led innovation, and contextually relevant AI solutions that reflect the needs and priorities of African populations.

Ultimately, AI should not be viewed as a stand-alone technological solution to health inequities. Rather, its contribution to equitable healthcare will depend on responsible governance, inclusive innovation, meaningful stakeholder engagement, and sustained investment in health system strengthening. When implemented within these enabling conditions, AI has the potential to become an important catalyst for advancing universal health coverage and reducing health inequities across Sub-Saharan Africa.

## Supplementary Material

PRISMA_ScR Checklist_Tapera et al.docx

Supplimentary Appendix 2 Pubmed Database search query.docx

## Data Availability

All data generated or analysed during this study are included in this published article and its supplementary information files. The complete electronic search strategies for all databases are provided in Supplementary Appendix 2 to support the transparency and reproducibility of the review.
