# Supplementary material for: Between the hype and harm: does artificial intelligence in health offer solace or further exclusion for marginalised populations in Sub-Saharan Africa? A scoping review
Source: Glob Health Action. 2026 Aug 3;19(1):2710547. doi: 10.1080/16549716.2026.2710547 (PMC13435285; doi:10.1080/16549716.2026.2710547)
Supplement: Supplementary file 1 [file ZGHA_A_2710547_SM8509.docx]

**Pubmed Database search query**

(("Artificial Intelligence"[Mesh]

OR "Artificial Intelligence"[tiab]

OR "Machine Learning"[tiab]

OR "Deep Learning"[tiab]

OR algorithm*[tiab]

OR "Neural Network*"[tiab]

OR "Predictive Model*"[tiab])

AND

("Health"[Mesh]

OR healthcare[tiab]

OR "Digital Health"[tiab]

OR mHealth[tiab]

OR eHealth[tiab]

OR "Clinical Decision Support"[tiab])

AND

("Health Equity"[Mesh]

OR equity[tiab]

OR inequity[tiab]

OR inequality[tiab]

OR marginali*[tiab]

OR vulnerable[tiab]

OR exclusion[tiab]

OR discrimination[tiab]

OR bias[tiab]

OR justice[tiab]

OR stigma[tiab]

OR PROGRESS-Plus[tiab])

AND

("Africa South of the Sahara"[Mesh]

OR "Sub-Saharan Africa"[tiab]

OR SSA[tiab]))

**Limits**

Language: English

Publication years: 2020–2026
